# Biomarkers for early complications post hematopoietic cell transplantation: Insights and challenges

**DOI:** 10.3389/fimmu.2023.1100306

**Published:** 2023-02-02

**Authors:** Balaji Balakrishnan, Uday Prakash Kulkarni, Aswin Anand Pai, Raveen Stephen Stallon Illangeswaran, Ezhilpavai Mohanan, Vikram Mathews, Biju George, Poonkuzhali Balasubramanian

**Affiliations:** ^1^ Department of Integrative Biology, School of Bio Sciences and Technology, Vellore Institute of Technology, Vellore, India; ^2^ Department of Haematology, Christian Medical College, Vellore, India

**Keywords:** biomarker, GVHD, endothelial, SOS, HSCT

## Abstract

Hematopoietic cell transplantation is an established curative treatment option for various hematological malignant, and non-malignant diseases. However, the success of HCT is still limited by life-threatening early complications post-HCT, such as Graft Versus Host Disease (GVHD), Sinusoidal Obstruction Syndrome (SOS), and transplant-associated microangiopathy, to name a few. A decade of research in the discovery and validation of novel blood-based biomarkers aims to manage these early complications by using them for diagnosis or prognosis. Advances in this field have also led to predictive biomarkers to identify patients’ likelihood of response to therapy. Although biomarkers have been extensively evaluated for different complications, these are yet to be used in routine clinical practice. This review provides a detailed summary of various biomarkers for individual early complications post-HCT, their discovery, validation, ongoing clinical trials, and their limitations. Furthermore, this review also provides insights into the biology of biomarkers and the challenge of obtaining a universal cut-off value for biomarkers.

## Introduction

Hematopoietic cell transplantation (HCT) from matched related or unrelated donors to recipients with various hematological disease conditions has become a widely accepted curative treatment of choice. Especially with malignant hematological diseases, the graft versus tumor/leukemia effect (GVT) is a beneficial phenomenon expected to improve the outcome of the procedure. However, a similar effect where graft acting against the recipient’s cells, such as graft versus host disease (GVHD), leads to an undesirable outcome. Graft versus host disease (GVHD) still remains a predominant cause of morbidity and mortality in patients following HCT. Clinically GVHD may present as acute (aGVHD) or chronic (cGVHD) based on the symptoms and time of their presentation. The classical pathway of occurrence of GVHD includes damage of the target organs such as skin, eye, gastrointestinal (GI) tract, liver, or lung, followed by the release of a storm of cytokines, which increases the chance of the donor’s immunocompetent cells to recognize the host’s alloantigens (1). More than half of HCT patients develop GVHD. Although GVHD is treated by several immunosuppressive agents, responsiveness to these agents, GVHD related morbidity and mortality are still concerns that affect HCT outcomes greatly. In addition to GVHD, other serious complications include hepatic or pulmonary sinusoidal obstruction syndrome (SOS), opportunistic infections (bacterial, viral & fungal), and multiorgan damage. Attempts to improve HCT outcomes include predicting patients who are at high risk of developing post HCT complications, predicting their responsiveness to treatment and early diagnosis of these complications. Composite biomarkers of prognostic values have been recently used in confirming the diagnosis of some of these complications (2, 3).

Excluding the known likely causal factors for some of the adverse effects (such as the donor status, age, comorbidity, sex mismatch between donor and recipient, conditioning, and post-HCT immunosuppressive drug levels), various centers performing allogeneic HCT are concentrating on finding efficient, reliable and robust markers from biological fluids for informative, early detection or differential diagnosis of these complications to optimize the treatment as well as improving the outcome (4–6). Many have successfully reported a variety of blood plasma, serum, and fecal biomarkers, while only a handful of these is repeatedly tested and validated and likely to be used as a biomarker routinely (7). The biomarkers from these sources may be soluble factors, cellular markers, or genetic markers. While many candidate biomarkers from plasma were evaluated and verified in independent cohorts, multi-center clinical trials are still needed to validate their clinical applicability. Similarly cell-free DNA have also been recently evaluated for identifying an array of post-HCT complications including aGVHD, relapse, infection, engraftment failure and chimerism status with an objective of employing a single test/technique for elucidating a comprehensive panel of post-HCT complications (8).

However, one of the major limitations of these biomarker studies is the varying cut-off value as a reference to predict or diagnose these complications between centers. Moreover, and not all biomarkers are referenced across the normal cut-off values between healthy individuals and patients undergoing transplantation. Often these biomarkers are tested between HCT patients with and without complications. This review provides insights into the biological significance of biomarkers, their discovery and validation for HCT complications, challenges in quantification or techniques, and lack of universal target cut-offs.

## The biological significance of biomarkers

Plasma biomarkers have been extensively evaluated for complications post HCT, since classic clinical risk scores such as HCT related co-morbidity index often fail to predict, diagnose or prognose such complications. The ultimate aim of plasma biomarker evaluation is its clinical translatability in predicting HCT complications, their severity and their response to treatment. On the other hand understanding the biology of these biomarkers would also pave way for developing more rational and effective treatment strategies for HCT complications. However, literature on biology of the biomarkers for HCT complications appear scanty. While increasing evidences suggest endothelial injury as a common cause for most HCT complications, a complete understanding is still lacking. Here we review the biology of a few biomarkers which are extensively evaluated for multiple overlapping complications.

## ST2

The suppression of tumorigenicity 2 (ST2) is a receptor belonging to the interleukin (IL)-1 family and binds specifically to IL-33. ST2 is present in two isoforms: a transmembrane form and a soluble form. The membrane-bound ST2 receptor is expressed on various hematopoietic cells such as T helper 2 (Th2) cells, natural killer (NK) cells, mast cells, antigen-presenting cells, and regulatory T cells (Tregs) (9, 10). The IL33/ST2 complex signaling in these cell types has been observed to have proinflammatory and anti-inflammatory responses depending on the disease type (11–13). During acute GVHD, a surge in IL33 has been observed both in the clinical scenario and in mice models of alloHCT. The mucosal barrier tissues, such as the skin, gastrointestinal tract, and liver, have been significant sources of IL33. During the alloHCT conditioning regimen, damage to these tissues increases IL33 production/release that drives donor Th1 cells expansion leading to inflammatory phenotype and further tissue injury. Recently, it was demonstrated that IL-33 acts directly on donor T cells and increases Tbet expression leading to enhanced Th1 cell polarization and expansion. However, despite these observations of elevated IL-33, this could not be used as a specific biomarker for aGVHD due to its pleiotropic effects.

**Figure 1:**
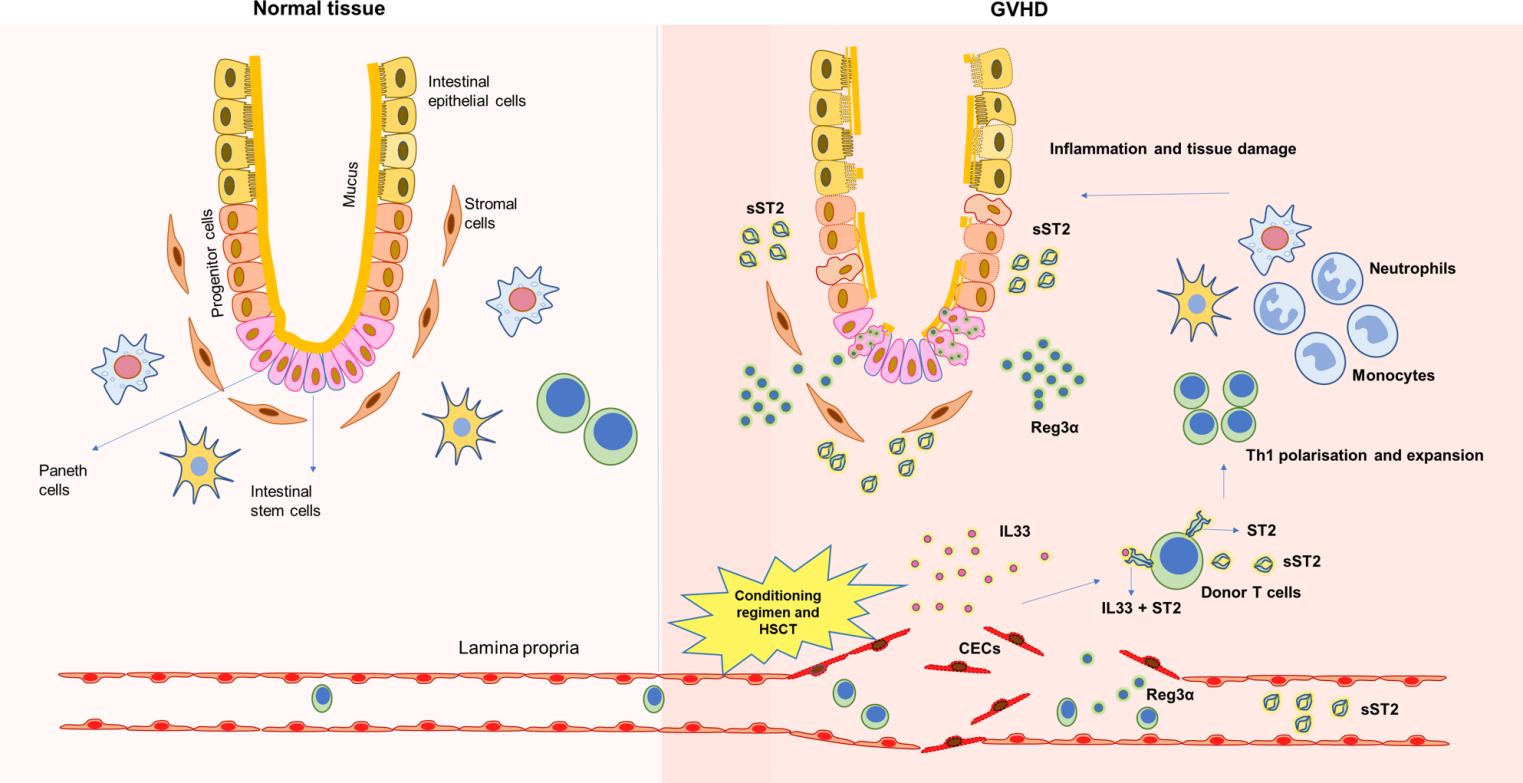
Underlying tissue damage during GVHD and release of soluble biomarkers. HSCT transplant procedures, including conditioning regimen, damage underlying endothelium, inflames the tissue and releases soluble factors that could be used as biomarkers during GVHD.

The soluble ST2 (sST2) receptors are expressed in endothelial cells, epithelial cells, fibroblasts, and T cells (14). The soluble ST2 act as decoy receptors, sequestering free IL-33, thereby preventing IL-33-mediated proinflammatory actions (15, 16). Thus, sST2 was generally considered to negatively regulate IL-33 function (**Figure 1**). However, this contradicts the association of elevated sST2 with GVHD severity in patients. A possible explanation given by earlier studies was that the release of sST2 in the serum occurs very late in the inflammatory response resulting in the inability of sST2 to sequester circulating IL33 (17).

Zhang J et al. demonstrated in a minor mismatch GVHD model and xenograft GVHD model that sST2 was secreted by intestinal stromal cells, endothelial cells, and alloreactive T cells. More importantly, as GVHD progresses, it was shown that pathogenic T cells (Th17 and Tc17) secrete more sST2 and express less mST2, thereby correlating elevated plasma sST2 levels during alloreactivity. Transient blockade of sST2 during GVHD increased Th2 transcription factor GATA3 and cytokine IL-4, improving Th2 phenotype, which protects against severe GVHD (18).

An overall picture of the ST2/IL33 axis in a severe GVHD context remains elusive. Whether sST2 is involved in the pathophysiology of GVHD or it is just a circulating biomarker indicating GVHD severity remains to be clarified.

## Reg3α

Regenerating islet-derived -3 α (Reg3α) alpha is one of the antimicrobial peptides secreted by Paneth cells of the gastrointestinal tract and is a C-type lectin having bactericidal actions on most gram-positive bacteria (19). The crypts’ innate lymphoid cells 3 (ILC3) secrete IL22, which induces Paneth cells to secrete Reg3α (**Figure 1**) (20). During HSCT, the crypt cells, including the Paneth cells, are damaged; hence, their numbers are inversely associated with GVHD severity (21). GVHD-induced damage to the gastrointestinal crypt and intestinal mucosa decreases IL22 production and releases antimicrobial peptides stored in these cryptic cells into the bloodstream. Thus, the increased plasma level of Reg3α was strongly associated with GI-GVHD enabling their use as a biomarker (22). It was also observed *in-vivo* in mice models of GVHD that the progression of GVHD suppresses Reg3γ (mouse homolog of human Reg3α) in the GI tract, further worsening GVHD. However, administration of IL22 has been shown to protect the crypt from damage, thereby preventing Reg3γ from being released into circulation. Mechanistically it was demonstrated that Reg3γ functions as an anti-apoptotic protein for intestinal stem cells (ISCs) and Paneth cells (23). Thus, Reg3α has the dual role of being an antimicrobial peptide as well as a survival signal preventing apoptosis of ISCs and Paneth cells.

While Il22 from host cells was recognized to promote intestinal stem cell survival and suppress GI-GVHD (24), a few studies have also shown that IL22 from donor cells augments GI-GVHD (25, 26). In a mouse model of steroid-refractory GVHD, by Song Q. et al. demonstrated that IL22 was produced by donor Th/Tc22 cells, leading to excess production of Reg3γ. However, such excess Reg3γ was shown to result in dysbiosis and worsening of GVHD. Thus, REG3γ could be a therapeutic target for treating steroid-refractory GVHD (27). Hence, whether Reg3alpha is a therapeutic target or a biomarker remains an enigma.

## TIM3

T cell immunoglobulin and mucin domain 3 (TIM3) is a transmembrane receptor protein expressed on interferon γ producing T cells, Tregs, myeloid cells, natural killer cells, and mast cells (28). The primary function of TIM3 is to inhibit Th1 responses and cytokine expressions. Hence, its dysregulation correlates with most autoimmune diseases, such as multiple sclerosis (29) and type I diabetes (30). Increased expression of TIM3 has been observed in solid tumors such as lung cancer, gastric cancer, colon cancer, etc., and their high expression levels were associated with low overall survival (31).

Elevated levels of TIM3 in the plasma of patients with GVHD (32) and osteosarcoma (33) have been observed, facilitating their use as potential biomarkers. However, the mechanism of soluble TIM3 release remains an enigma. It could be a splice variant, a metalloproteinase-dependent cleaved product, or a soluble fragment from apoptotic cells. While soluble TIM3 was found to express as a splice variant in mice splenocytes (34), their existence in humans is still debatable.

The mechanistic understanding of TIM3’s action in aGVHD remains incomplete and is not explored much. Oikawa et al. demonstrated in a murine model of GVHD that TIM3 plays a crucial role in the activation of CD8+ T cells, which are the primary effectors in target organ destruction in aGVHD. Two weeks post-transplantation, the CD8+ T cells in the spleen and liver of GVHD mice showed enhanced TIM3 and interferon γ (IFNγ) expression. Moreover, the CD8+T cell infiltration was dominant in the liver of GVHD mice (35). However, the exact mechanism of TIM3 induction in these cells and their shedding into peripheral circulation remains unclear.

## Elafin

Elafin is a biomarker associated with the diagnosis and prognosis of skin GVHD. It is an epithelial protein secreted by keratinocytes in response to inflammatory cytokines. Hence elafin’s expression is higher in the inflamed epidermis and absent/low in the normal epidermis. It is a peptidase inhibitor-3 or skin-derived anti-leukoprotease (SKALP) with antimicrobial activity and priming innate immune responses (36). It was observed that when induced by GVHD mediating inflammatory cytokines, human keratinocytes express elafin significantly (37, 38). However, the mechanism by which elafin from keratinocytes is released into circulation during GVHD remains unclear.

## Biomarkers for acute GVHD

Biomarkers for acute GVHD have been extensively evaluated over the past decade in multiple HCT centers worldwide. These range from plasma, cellular, genetic and in a few cases, a combination. Biomarkers for aGVHD have been measured pre-HCT to personalize GVHD prophylaxis, post-HCT before or at the onset of GVHD, to confirm the diagnosis of GVHD or after treatment to predict treatment response.

### Biomarkers measured pre-transplant for modulating GVHD prophylaxis

CD86 is the ligand for costimulatory (CD28) and coinhibitory (CTLA-4) molecules. Karaban et al. reported that the recipients’ CTLA-4 CT60GA[GG] genotype, myeloablative conditioning regimen, and use of an unrelated donor were independent predictors of acute GVHD (39). Also, the same group has shown that donor and recipient CTLA-4 mRNA and recipient membrane protein expression measured before transplantation are prognostic for acute GVHD (40). Later they also reported a lack of association of CD86 gene polymorphisms with GVHD. However, they noted a gene-gene interaction wherein patients with a specific CD86 genotype and a CTLA-4 genotype was associated with an increased risk of aGVHD. With a combination of specific donor CD86 genotype and recipient CTLA-4 genotype there was an elevated GVHD risk (41).

In a study exploring the role of donor genetic variations in glucocorticoid pathway on steroid responsiveness of GVHD, although donor SNPs in *ZAP70* and *DUSP1* genes were associated with response, these were not statistically significant on adjustment for multiple testing (42). Cytokine biomarkers – TLR4 and TNFR1 are significantly increased in steroid-refractory acute GVHD compared to those with steroid-responsive GVHD (43).

DNAX accessory molecule-1 (DNAM-1, or CD226) is a leukocyte adhesion molecule constitutively expressed on most CD4+ T cells, CD8+ T cells, natural killer (NK) cells, and monocytes. A retrospective study from Japan showed that higher soluble DNAM-1 measured between day -7 to day 0 of an allotransplant was predictive of a higher risk of acute GVHD. Using a cut-off of 30pM for soluble DNAM-1, the sensitivity for predicting acute GVHD was 43.8%, while the specificity was 82.6% (44). Serum IL-6 levels measured pre-conditioning and one week after transplant were predictive of acute GVHD and transplant-related mortality (45), although, the pleiotropic nature of IL-6 may be a concern. Specific donor graft characteristics like an elevated proportion of T cells with low CD127 and high PD-1 expression have been associated with subsequent acute GVHD (46).

### Biomarkers measured post-transplant before or at GVHD onset

Specific patterns of immune reconstitution following transplantation, such as increased CD8+ T cells (both naïve and memory) in the early post-transplant period (on day 15), have been associated with development of subsequent acute GVHD (47).

Elevated plasma REG3α measured at the onset of GVHD predicted non-response to treatment at 4 weeks and also 1 year non-relapse mortality (22). Elevated plasma elafin at the onset of skin GVHD is associated with higher maximum grade of GVHD and also non-relapse mortality (48). Also, elevated ST2 predicts steroid resistance in acute GVHD and non-relapse mortality (4). In non-myeloablative transplants, elevated plasma ST2, REG3α, and elafin measured early post-transplant were predictive of acute GVHD (49). Similarly, in T-replete haplotransplants, elevated plasma ST2 and REG3α measured early post-transplant were predictive of acute GVHD and non-relapse mortality (50). In patients undergoing matched donor T deplete transplantation using anti-thymocyte globulin or alemtuzumab, a biomarker panel including HGF, elafin, sIL-2Rα, sTNFR1, and REG3α was predictive of GVHD and its severity (51).

Lower serum sIL-27Rα at the time of neutrophil engraftment is predictive of acute GVHD and has been shown to correlate with other serum GVHD biomarkers (52). Elevated plasma levels of sIL2-Rα and TIM-3 in the early post-transplant period predicted increased transplant-related mortality and acute GVHD (53).

Similarly, expression patterns of genes and a few microRNAs have also been evaluated as biomarkers post HCT. Transcripts levels of *FOXP3*, *ICOS*, *CD52*, and *CASP1* genes involved in alloreactive immune responses and immune cell interactions were predictive of acute GVHD using a personalized modeling-based gene selection (PMGS) method (54). A risk score developed using metabolite and transcriptome analysis incorporating 5 metabolite markers from glycerophospholipid metabolism was predictive of acute GVHD (55). miR-155 and miR-146a measured in target tissues at the time of GVHD onset and measured in extracellular vesicles in serum and urine in the early post-transplant period before GVHD onset have been predictive of acute GVHD (56).

### Biomarkers measured at symptom onset for supporting the diagnosis of GVHD (elafin, calprotectin)

Fecal, and not serum calprotectin is a biomarker for acute gut GVHD and can potentially help diagnose gut GVHD (57). Also, low tissue amphiregulin expression on immunohistochemistry has been reported in 74% of patients with acute gut GVHD and might aid in diagnosis without classic apoptotic changes (58). Similarly, tissue, and not plasma, elafin on immunohistochemistry can aid in diagnosing acute GVHD involving the skin (59, 60).

Analysis of exosomal miRNA expression using quantitative RT-PCR on plasma samples showed that miR-128 was elevated in late-onset GVHD and is a promising diagnostic marker of late-onset GVHD (61). However, the turnaround times for these biomarkers may limit their practical utility.

### Biomarkers measured at the onset of GVHD and after treatment of GVHD for potentially predicting response

The Mount Sinai Acute GVHD International Consortium (MAGIC) algorithm probability score (MAP score) based on plasma ST2 and REG3α is a response biomarker for acute GVHD. After four weeks of therapy, it was shown to predict non-relapse mortality better than the change in clinical symptoms. The MAP score was predictive of non-relapse mortality within every clinical grade of acute GVHD (62). The MAP score has been shown to be helpful when measured at day 28 along with the disease risk index could also identify patients at high relapse risk and low non-relapse mortality risk who can potentially benefit from strategies to enhance the graft versus leukemia effect for relapse prevention (63). Rising REG3α following treatment for GVHD using a novel combination of upfront steroids+ruxolitinib was shown to be a predictor of refractory GVHD (64). However, there is no prospective clinical study on biomarker-based intervention for adding second-line therapy for acute GVHD. A list of various biomarkers measured at different stages during HCT procedure and/or at GVHD onset, with potential clinical values that could help in prediction, diagnosis or prognosis for acute GVHD is summarized in **Table 1**.

**Table 1:** List of various types of biomarkers with clinical values that could help in the prediction, diagnosis, or prognosis for Acute GVHD.

S.No	Biomarker	Type	Biological Specimen(s)	Detection Method(s)	Biomarker level(s)	Clinical Value	Ref(s)
1	CD86/CTLA4 polymorphisms	Immunological	DNA	Genotyping	Polymorphism	Predictive	(12)
2	DNAM-1/CD226	Immunological	Serum	ELISA	Increased levels	Predictive	(9)
3	IL-6	Immunological	Serum	ELISA	Increased levels	Predictive	(13)
4	CD127/PD-1	Immunological	Peripheral Blood	Flow cytometry	High frequency of PD-1^+^ T cells and low frequency of CD127^+^ T cells in donor graft associated with grades II-III aGVHD	Predictive	(14)
5	*FOXP3* and *ICOS*	Immunological	Peripheral Blood	RT-PCR	Low levels associated with aGVHD Increasing levels correlate with response to anti-GVHD therapy	Diagnostic /Prognostic	(21)
6	PAF, LysoPC, PE, PC, and LysoPE	Metabolic (Biochemical)	Plasma	LC-MS	aGVHD risk score developed	Predictive /Prognostic	(22)
7	sIL2Ra	Immunological	Plasma/Serum	ELISA	Decreased levels	Prognostic	(23)
8	miR-146a and miR-155	MicroRNA	Plasma/Serum	RT-PCR	Increased expression	Diagnostic /Prognostic	(24)
9	Calprotectin	Immunological	Serum	ELISA	Increased levels	Diagnostic /Prognostic	(26)
10	Amphiregulin	Immunological	Serum	ELISA	Decreased levels	Diagnostic /Prognostic	(27)
11	Exosomal miR-128	MicroRNA	Plasma	RT-PCR	Increased expression	Diagnostic Biomarker for Late-Onset aGVHD	(30)
12	Elafin	Epidermal	Tissue	Immunohistochemistry	Increased expression	Diagnostic	(28, 29)
13	Donor *ZAP70* and *DUSPI* SNPs	Biochemical	DNA	Genotyping	Polymorphism	Prognostic	(31)
14	TLR4 and TNFR1	Immunological	Serum	ELISA	Increased levels	Prognostic	(32)
15	ST2, REG3α and Elafin	Immunological	Plasma	ELISA	Increased levels	Predictive/Prognostic (Non-myeloablative HCT setting)	(17)
16	REG3α and Elafin	Immunological	Plasma	ELISA	Increased levels	Predictive/Prognostic (Myeloablative HCT setting)	(16)
17	ST2 and REG3α	Immunological	Plasma	ELISA	Increased levels	Predictive/Prognostic (Haploidentical HCT setting)	(18)
18	HGF, Elafin, sIL-2Rα, sTNFR1, and REG3α	Immunological	Plasma	ELISA	Increased levels	Predictive/Prognostic (Matched T-cell deplete HCT setting)	(19)

CD, Cluster of Differentiation; CTL14, cytotoxic T-lymphocyte–associated antigen 4; HGF, Hepatocyte growth factor; miRNAs, microRNAs; DNAM1, DNAX accessory molecule-1; IL-6, Interleukin-6; PD-1, Programmed Cell Death Protein 1; FOXP3, forkhead box P3; ICOS, Inducible T-cell COStimulator; PAF, Platelet-activating factor; LysoPC, Lysophosphatidylcholines; LysoPE, Lysophosphatidylethanolamine; PC, Phosphatidylcholine; PE, Phosphatidylethanolamine; LC-MS, Liquid Chromatography-Mass Spectrometry; ZAP70, Zeta Chain Of T Cell Receptor Associated Protein Kinase 70; DUSP1, Dual specificity protein phosphatase 1; TLR4, Toll-like receptor 4; TNFR1, Tumor necrosis factor receptor 1; ST2, soluble suppressor of tumorigenicity 2; REG3a, regenerating islet-derived protein 3a; sIL2Rα, soluble interleukin-2 receptor alpha-chain; ELISA, Enzyme-linked immunosorbent assay; RT-PCR, Reverse transcription–polymerase chain reaction.

## Biomarker guided pre-emptive therapy for GVHD

Initial discovery and validation of ST2 as a biomarker for aGVHD also led to studies investigating inhibitors for ST2 in animal models (65). While this is still in progress, biomarker evaluation has progressed towards guided therapy/intervention for aGVHD with already existing anti-GVHD strategies. Gergoudis et al. have recruited patients at high risk for developing steroid-refractory GVHD (SR-GVHD) based on the MAGIC algorithm probability (MAP) scores on days 7 and 14 post-HSCT. These patients were then treated with alpha-1 anti-trypsin (AAT), a serine protease inhibitor with proven activity against GVHD. Although AAT treatment was well tolerated, the incidence of SR-GVHD was not lowered (66). Nevertheless, the power of biomarker-based SR-GVHD prediction could not be undermined. Instead, such studies pave the way for investigating more treatment options. A recent study involving a prospective phase 2 trial stratified patients based on sIL-2Rα and IL-15 levels. High-risk patients (sIL-2Rα 4500 ng/L or IL-15 31 ng/L) were treated with rabbit anti-thymocyte globulin (ATG) 3 mg/kg on day 8 post-transplant and were compared with controls who had the biomarkers measured but did not participate in this interventional trial. A reduction in GVHD was observed in these patients compared to high-risk controls who did not receive ATG (Hazards ratio of 0.48), signifying the feasibility and effectiveness of such an approach (67).

## Biomarkers for sinusoidal obstruction syndrome

Sinusoidal Obstruction Syndrome (SOS), previously, known as veno-occlusive disease, is a severe complication post HSCT affecting liver sinusoidal endothelial cells. About 13 to 20% of allogeneic HSCT recipients develop SOS and the severe form of SOS is associated with multiorgan failure and mortality (68).

Typically, SOS has been observed between one to three weeks post-HSCT. Often clinically indistinguishable from other causes of weight gain, ascites, abdominal pain, and jaundice.

Factors such as conditioning regimen drugs or radiation, releasing cytokines from injured tissues, and the endogenous microbial substances that cross the compromised mucosal barriers lead to the activation of sinusoidal endothelial cells. Sustained activation can progress to endothelium damage (69). The sinusoidal endothelial cells swell and round, forming gaps in the sinusoidal barrier. These alterations facilitate the egress of leucocytes, RBCs, and cellular debris into the perisinusoidal space beneath the endothelial cells and disrupt the endothelial lining leading to sinusoidal embolisms and obstruction of the sinusoidal flow, liver dysfunction, ascites ultimately leading to multiorgan failure (70, 71).

Some reliable markers of endothelial activation and damage are soluble cellular adhesion molecules (sVCAM1, sICAM1, and sP-selectin), coagulation factors (Von Willebrand factor (VWF), thrombomodulin (TM) and plasminogen activator type-1 (PAI-1)) (**Table 2**).

**Table 2:** List of various types of biomarkers with clinical values that could help in the prediction, diagnosis, or prognosis for SOS.

S.No	Biomarker	Type	Biological Specimen(s)	Detection Method(s)	Biomarker level(s)	ClinicalValue	Ref(s)
1	Ferritin	Biochemical	Serum	Biochemical tests	Increased levels	Predictive	(72)
2	Uric Acid	Biochemical	Serum	Biochemical tests	Increased levels	Predictive	(73)
3	Liver Profiling	Physiological	Liver	MRI	Increased iron overload	Predictive/ Diagnostic	(74)
4	HGF	Immunological	Serum	Immunoassay	Increased levels	Predictive	(75)
5	GSTs GSTA1 GSTM1	Genetic	DNA	Genotyping	Polymorphism	Predictive	(76, 77)
6	MTHFR	Genetic	DNA	Genotyping	Polymorphism	Predictive	(78)
7	HPSE	Genetic	DNA	Genotyping	Polymorphism	Predictive	(79)
8	sICAM-1	Endothelial	Plasma/Serum	ELISA	Increased levels	Prognostic/ Predictive	(69, 80–82)
9	sVCAM-1	Endothelial	Plasma/Serum	ELISA	Increased levels	Prognostic/ Diagnostic	(80)
10	sE-Selectin	Endothelial	Plasma/Serum	ELISA	Increased levels	Prognostic/ Predictive	(69)
11	sP-Selectin	Endothelial	Plasma/Serum	ELISA	Increased levels	Prognostic/ Predictive	(81, 83)
12	VWF	Endothelial	Plasma/Serum	ELISA	Increased levels	Prognostic	(69, 84)
13	TM	Endothelial	Plasma/Serum	ELISA	Increased levels	Prognostic	(69, 84)
14	PAI-1	Endothelial	Plasma/Serum	ELISA	Increased levels	Prognostic/ Diagnostic	(84–87)
15	VEGF	Endothelial	Plasma/Serum	ELISA	Increased levels	Predictive	(88)
16	ANG2	Endothelial	Plasma/Serum	ELISA	Increased levels	Prognostic/ Diagnostic	(82)
18	miRNAs	MicroRNA’s	Plasma/Serum	RT-PCR/Micro-seq Microarray	miRNA dependent	Prognostic	(89)
19	TNFa	Immunological	Plasma/Serum	ELISA	Increased levels	Prognostic	(90)
20	ST2	Immunological	Plasma/Serum	ELISA	Increased levels	Diagnostic	(82)
21	REG3a	Immunological	Plasma/Serum	ELISA	Increased levels	Prognostic	(80)
22	TIM3	Immunological	Plasma/Serum	ELISA	Increased levels	Prognostic	(80)
23	HA	Immunological	Plasma/Serum	ELISA	Increased levels	Prognostic/ Diagnostic	(82)
24	L-Ficolin	Immunological	Plasma/Serum	ELISA	Decreased levels	Diagnostic	(82)
25	sIL2Ra	Immunological	Plasma/Serum	ELISA	Increased levels	Prognostic	(90)
26	IGF-1	Immunological	Plasma	Immunoassay (Chemiluminescence)	Decreased levels	Predictive	(91)
27	EASIX	Biochemical	Panel (Serum/Blood)	Biochemical tests	Increased levels	Diagnostic	(92)

SOS, sinusoidal obstruction syndrome; HGF, Hepatocyte growth factor; GST, Glutathione S-transferases; MTHFR, Methylenetetrahydrofolate Reductase; HPSE, Heparanase; sICAM-1, soluble Intercellular CAM protein 1; sVCAM-1, soluble vascular CAM protein; VWF, Von Willebrand factor; TM, thrombomodulin; PAI-1, plasminogen activator type-1; VEGF, vascular endothelial growth factor; ANG2, Angiopoietin2; EV, extracellular vesicles; miRNAs, microRNAs; TNFa, tumor necrosis factor alpha; ST2, soluble suppressor of tumorigenicity 2; REG3a, regenerating islet-derived protein 3a; TIM3, T-cell immunoglobulin and mucin domain-containing protein 3; HA, hyaluronic acid; sIL2Ra, soluble interleukin-2 receptor alpha-chain; EASIX, Endothelial Activation, and stress index panel; IGF1, Insulin-like growth factor 1; ELISA, Enzyme-linked immunosorbent assay; RT-PCR, Reverse transcription–polymerase chain reaction; micro-seq, miRNAs sequencing.

The microenvironment of the endothelium is significantly altered in patients who undergo allo-HCT. Allo-HCT patients who develop SOS have a significant increase in both VWF and TM levels (69, 84). Furthermore, in patients receiving both tacrolimus and sirolimus as GVHD prophylaxis, levels of VWF and TM (together with ICAM-1 and E-selectin level) serve as SOS predictive biomarkers one-week post HCT (69). Two weeks post- HCT, plasma levels of REG3α, sVCAM1, sICAM1, and TIM3 are shown to be consistently elevated in patients who developed SOS (80). P-selectin levels are shown to be selectively higher in patients who develop severe SOS and elevated circulating levels of PAI-1 allow differential diagnosis between SOS and GVHD, as patients with SOS show elevated PAI-1 but not those with GVHD (81, 85, 86).

In a recent study, a composite diagnostic panel of three biomarkers: L-Ficolin, hyaluronic acid (HA), and VCAM-1, was reported to detect patients at high risk of SOS as early as the first day after HCT, even before clinical manifestation of SOS (82). Additionally, it was proposed that the biomarker panel ST2, ANG2, L-Ficolin, HA, and VCAM-1 could be helpful in the diagnosis of SOS (82). Inflammatory cytokines such as IL2, IL6, IL33, IFNγ, and TNFα are mediators of EC activation and damage. Both TNFα and soluble IL2 receptor α (sIL2Rα) are shown to be elevated during GVHD and SOS (90, 93, 94).

## Biomarkers for other early complications post-HCT

Promising results from the studies evaluating biomarkers for GVHD and SOS have also led to the identification of similar plasma biomarkers for other early HSCT complications, such as transplant-associated- thrombotic microangiopathy (TA-TMA) and engraftment syndrome (ES). TA-TMA is characterized by occlusion and disruption of microcirculation as a result of micro-thrombi deposition. It is believed that the disruption of microcirculation results from endothelial dysfunction. Lia G et al. reviewed that the endothelial dysfunction could be due to persistent insult to the endothelium caused throughout the HSCT procedure, starting from the conditioning regimen and subsequently through calcineurin inhibitors (95).

While there are multiple causes for endothelial injury, neutrophil extrusion traps (NETs) also appear to be one component evaluated in the TA-TMA context. A significantly elevated level of NETS, within the first 4 weeks post-HSCT, has been reported to be associated with an increased risk of TA-TMA (96). In contrast, the same study could not find a possible association of thrombomodulin (expressed by endothelial cells and serves as a cofactor for thrombin) with the occurrence of TA-TMA, indicating challenges in understanding the pathophysiology of TA-TMA. Interestingly, elevated ST2 levels on day 14 post-HSCT was also reported to be associated with TA-TMA. The clinical overlap between GVHD and TA-TMA occurrence and endothelial injury as a common factor for both conditions, indicates that ST2 could also be a possible biomarker for TA-TMA (97). A recent study by Okamura H et al. has shown that elevated levels of complement factor Ba on day 7 post-HSCT significantly predicted TA-TMA (98).

Similarly, the symptoms of engraftment syndrome (ES) post HCT appears overlapping with that of either GVHD or with infections. Biomarkers that could help in the early differential diagnosis of ES from other conditions with overlapping symptoms could improve HCT outcomes. Procalcitonin (PCT), a hormokine has been reported to be elevated in ES patients that could possibly be used as biomarker (99). Knoll et al., reported procalcitonin levels 2ng/ml could possibly distinguish patients with ES from patients with bacterimia (100). However, since PCT is also a FDA approved biomarker for sepsis and febrile neutropenic pateints with infections (101, 102), the use of PCT for ES needs to be evaluated in multiple cohorts.

Most of the complications post HCT appears to be as a result of persistent insult to the damaged endothelium throughout HCT procedures. Hence many markers of endothelium damage have been extensively evaluated as potential biomarkers for most HCT complications as well (103, 104).

## Challenges in evaluating biomarkers for post-HCT complications

More than a decade of progress in discovering and validating biomarkers for HSCT complications led to incorporating them in clinical trials to verify their impact as a diagnostic, prognostic, or tool for pre-emptive therapy/intervention. For example. Reg3α was shown to distinguish diarrhea due to GI-GVHD from diarrhea due to non-GVHD causes (22). In contrast, Elafin could distinguish skin GVHD from drug hypersensitivity rashes (DHR) (ref). Also, REG3α and elafin were shown to distinguish diarrhea and rashes due to a more systemic disease than GI-GVHD alone (105). Similarly, ST2, TIM3, and IL6 were shown to be diagnostic biomarkers for aGVHD (106). However, these biomarkers’ prospective utilization has not yet been achieved. There are more challenges in translating biomarker concentrations toward a possible clinical decision in terms of diagnosis or intervention:

## Determination of cut-off values

Plasma biomarker concentrations need a range of cut-off values to make clinical decisions. Different HCT centers have evaluated various biomarkers either singly or as a panel. However, there appear to be no universal cut-off valuesfor different biomarkers, probably due to the methods employed to derive cut-off values. For instance, Hartwell et al. developed an algorithm using logistic regression analysis of biomarker concentrations to derive cut-off values (6). Other groups have used individual biomarker concentrations in respective cohorts to derive cut-off values (4).

Similarly, the association of biomarkers towards specific HSCT outcomes that could not be verified in different cohorts precludes deriving a universal cut-off values. For instance, it was shown that high ST2 levels correlated with steroid-refractory GVHD (4) but was subsequently shown in different cohorts to be associated with six months of non-relapse mortality and not with GVHD (107, 108). Various groups have reported different cut-off values for the same biomarker [For example, ST2: 33.9 ng/ml (107); 740 pg/ml (4); 3230 ng/ml (50), REG3α: 151 ng/ml (22); 1989 pg/ml (50)]. Finally, there always appears an overlap in biomarker concentrations in cohorts with and without HSCT complications impedes a universal cut-off values derivation. Thus, establishing a universal reproducible cut-off values remains a challenge.

## Single biomarker vs. panel

Due to the overlap in concentrations of biomarkers in patients with and without HSCT complications, many groups have reported that a single biomarker could be of little value correlating with HSCT outcomes as opposed to a biomarker panel. Elafin was initially discovered to be associated with skin GVHD (48). However, its utility appears very limited owing to the lack of reproducibility (60). The inclusion of elafin to REG3α and ST2 was also shown to be of little value in improving the accuracy of assessing HSCT outcomes (109). On the other hand, biomarkers such as ST2 and REG3α are potentially promising as single biomarkers correlating with therapy-resistant GVHD and GI-GVHD and as panels predicting six months of non-relapse mortality (NRM) (4). A special consideration towards the sensitivity and specificity of biomarkers, either as single or panel, needs to be given to biomarkers’ clinical translatability.

Beyond these challenges, the time points for biomarker testing and the frequency of such testing are also not standardized. There is considerable variation in these parameters in the reported literature so far.

## Conclusion

Non-invasive biomarkers have been comprehensively evaluated for detection, diagnosis, and/or prognosis of early complications post-HCT in multiple centers. Various studies have evaluated individual biomarkers alone or as a panel towards GVHD. The past decade of voluminous data has shown that biomarker panels, as opposed to individual biomarkers, are more valuable in diagnosing GVHD or predicting GVHD severity. In this context, MAGIC, a GVHD biomarker panel employing an algorithm using logistic regression, appears to be so promising in terms of its clinical translatability since multiple centers have verified this. On the other hand, biomarkers for other complications, such as SOS, TA-TMA, etc., still need to be confirmed in multiple clinical settings. The association of endothelial damage with post-transplant complications has been a promising addition to the arsenal of biomarkers. However, biomarkers based on endothelial damage are greatly influenced by many factors, such as underlying disease, conditioning regimen, and post-transplant conditions. Nevertheless, multiple studies have progressed well in evaluating endothelial damage biomarkers toward post-HCT complications. EASIX panel to predict SOS severity is the best example.

Prospective studies and clinical trials incorporating biomarker based interventions with clinical endpoints are required to further evaluate the clinical translatability of these biomarkers. In absence of such studies being reported, the clinical translation of biomarkers in HCT is not ready for prime time. The longer turn-around times, variable cut-offs, and assay variabilities also remain as barriers towards practical utility of such biomarkers in HCT for clinical decision making and strategies to circumvent these are needed.

Equally important is understanding the biology of the hitherto validated biomarkers, which will have advantages such as guided pre-emptive therapy, finding novel therapeutic targets for HCT complications, and, more importantly, allowing us to validate if the biomarkers are sensitive and specific.

Progress in biomarker evaluation towards HCT complications is accompanied by challenges such as the derivation of a universal cut-off point, evaluation of individual or panel of biomarkers, and prospective biomarkers assessment. These challenges could be due to differences in techniques used to analyze biomarkers, and serum/plasma sample processing, including dilutions, conditioning regimen intensity, and the source of graft. Nevertheless, recently many studies are moving towards using biomarkers as a guide for preemptive therapy. Thus our knowledge of biomarkers for early complications is ever-expanding, leading to more significant progress in its clinical translatability.

## Author contributions

BB, UK, PB, designed the review structure and wrote the manuscript. EM, AAP and SI wrote the manuscript. BG and VM contributed to analysis and review of the manuscript. All authors contributed to the article and approved the submitted version.
